# Association of dietary fatty acids and the incidence risk of cardiovascular disease in adults: the Tehran Lipid and Glucose Prospective Study

**DOI:** 10.1186/s12889-020-09824-w

**Published:** 2020-11-19

**Authors:** Parvin Mirmiran, Zeinab Houshialsadat, Zahra Bahadoran, Sajad Khalili-Moghadam, Farhad Sheikholeslami, Fereidoun Azizi

**Affiliations:** 1 Nutrition and Endocrine Research Center, Research Institute for Endocrine Sciences, Shahid Beheshti University of Medical Sciences, P.O.Box 19395-4763, No. 24, Sahid-Erabi St, Yemen St, Chamran Exp, Tehran, Iran; 2 Prevention of Metabolic Disorders Research Center, Research Institute for Endocrine Sciences, Shahid Beheshti University of Medical Sciences, Tehran, Iran; 3 Endocrine Research Center, Research Institute for Endocrine Sciences, Shahid Beheshti University of Medical Sciences, Tehran, Iran

**Keywords:** Cardiovascular events, Fatty acids, Saturated fatty acid, Polyunsaturated fatty acid, Monounsaturated fatty acid, Oleic acid

## Abstract

**Background:**

Considering the inconsistent available findings regarding the cardioprotective effect of dietary fatty acid composition, we prospectively examined the feasible association between the dietary fatty acids and the cardiovascular disease (CVD) incidence in framework of the population-based Tehran Lipid and Glucose Study.

**Methods:**

A total of 2369 participants (19–70 years, 43.5% men) without CVD at baseline (2006–2008) were included and followed-up for 6.7 years. Fatty acids’ dietary intake was estimated using a 168-item semi-quantitative food frequency questionnaire. The CVD incidence risk across tertiles of dietary fatty acids was predicted via Cox proportional hazards regression models.

**Results:**

The average age and body mass index of the included population were 38.5 ± 13.3 years and 26.6 ± 4.8 kg/m^2^ at baseline. Over 6.7 years of follow-up, 79 cases of CVD were detected. The risk of CVD was lower in upper tertile of monounsaturated fatty acids, oleic acid, and docosahexaenoic acid + eicosapentaenoic acid among the tertiles. No significant associations were found between total fat, saturated and polyunsaturated fatty acids’ intake, and CVD.

**Conclusions:**

Our results suggest that the dietary fatty acid composition might affect the incidence risk of CVD within the Iranian population.

## Background

Cardiovascular diseases (CVD), which carry high psychological and medical impact as well as economic burden, has emerged as the leading factor of global morbidity and mortality, claiming 17.3 million lives each year [1]. Several modifiable and unmodifiable factors contribute to the risk of CVD [2]. It’s believed that the burden of CVD-related complications associated with dietary factors has been on rise over the past two decades [3]. Evidence regarding the association between fat consumption and the risk of CVD-related complications is controversial, and the fatty acid composition and the source of fat are critically influential on such inconsistencies [4, 5].

Several studies have previously investigated the association between dietary fatty acids and CVDs, most of which reported controversial results. In this regard, polyunsaturated (PUFA) and monounsaturated (MUFA) fatty acids-enriched dietary patterns were associated with lower CVD mortality and morbidity [6, 7]. Some studies argue that the increasing trend of CVD-related complications is due to increased consumption of trans-fat and saturated fatty acid (SFA) [6, 7]; however, there are studies that suggest an inverse association between SFA intake and stroke mortality [8, 9]. Therefore, reduced intake of dietary fat and SFA does not necessarily decrease the risk of CVDs, whereas replacing SFA with PUFA and MUFA certainly reduces the total cholesterol (TC) to high-density lipoprotein cholesterol (HDL-c) ratio and low-density lipoprotein cholesterol (LDL) concentrations to different extents [9]. Several studies have evaluated the cardioprotective properties of the fatty acid composition, but evidence from large-scale cohort studies to support this claim are limited [10].

The observed inconsistency can be attributed to participants’ characteristics, which has resulted in unclear conclusions in population-based studies. The importance of addressing the CVD dietary-related risk factors calls for a full clarification and firm conclusion on this matter. Also, the global dietary guidelines developed by the international health institutions require reconsideration in light of these findings; meanwhile, there should be clear recommendations in these guidelines. Therefore, the current study aimed to assess whether dietary fatty acid composition can predict future CVD events.

## Methods

### Study population

This study discussed the fatty acid composition in association with the CVD incidence risk in the prospective Tehran Lipid and Glucose Study (TLGS) framework [11, 12]. The TLGS is an ongoing investigation aimed to prevent non-communicable diseases (NCDs), reduce the NCD-associated risk factors, and promote a healthy lifestyle. Phase one of the TLGS took place in 1999 with 15,005 participants (≥ 3 years old), selected via the multistage cluster random sampling from a sample of residents in District No. 13 of Tehran, whose collected data were pooled together [11]. The measurements were repeated triennially for the assessment of any changes made to the risk of NCDs.

**Fig. 1:**
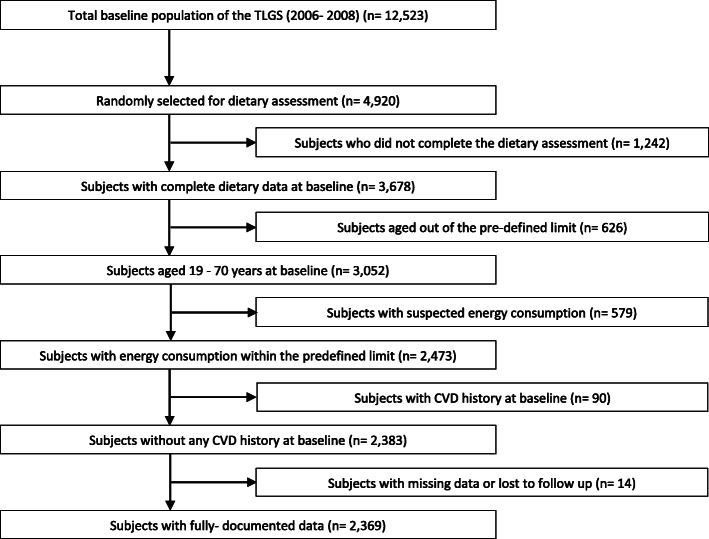
Flowchart of the study population

Figure 1 illustrates the successive steps involved in participants’ selection and recruiting. This study is based on the data collected during phase III of the TLGS (2006–2008 to 2014). During this phase, a total of 12,523 adult men and women completed the examinations. In total, 4920 subjects who were randomly selected completed the dietary assessment. The randomization was performed to minimize the time and cost involved in conducting the required data collection as well as avoiding the complexities associated with dietary data collection in large populations. Ultimately, the dietary results were obtained for 3678 individuals, which resulted in a response rate of 30%. By the exclusion of participants under 19 and over 70 years of age (*n* = 626), with suspected energy consumption (800 > x > 4200 kcal/d) (*n* = 579), CVD history at baseline (myocardial infarction, stroke, angina, coronary revascularization) (*n* = 90), and those with missing data or loss to follow-up (*n* = 14), a total of 2369 adults (1030 men and 1339 women) were recruited.

During the follow-up, the response rates for in-person visits and annual telephone call follow-ups were ranging from 55 to 75% and 80 to 90%, respectively. The participants were 19 to 70 years, with a mean age of 58.4 ± 9.7 and 37.4 ± 12.8 for those with CVD and no CVD, respectively (*P* = 0.001).

The present study specifically considered individuals with complete dietary, demographic, biochemical, and anthropometric data from phase three of the TLGS examinations, who have completed the Food Frequency Questionnaire (FFQ) at baseline and were not following any specific diets. Participants who have completed the FFQ shared similar baseline characteristics with the total population of phase three of the TLGS.

### Demographic and anthropometric measurements

Demographic information was collected through interviews with participants via standardized questionnaires. The interviews were conducted by a group of trained interviewers. Variable measurements of the TLGS were described in detail elsewhere [12]. The anthropometric measurements, including weight and height, were obtained via digital scales to the nearest 0.1 kg and ‘drop-down’ tape meters to the nearest 0.5 cm, respectively. The waist circumference (WC) was taken to approximately the nearest 0.1 cm with no pressure to the body. Diastolic and systolic blood pressures (SBP and DBP) were taken following a 15-min rest by a standard mercury sphygmomanometer, which was calibrated by the Iranian Institute of Standards and Industrial Researches. The final blood pressure was considered as the mean of the two measurements.

### Dietary assessment

The required demographic, dietary, biochemical, and anthropometric data were evaluated at the baseline. The typical dietary habit of the participants was evaluated by a validated 168-item semi-quantitative FFQ in the primary examination [13]. The reliability and validity of the questionnaire have been demonstrated in a random sample using FFQs [13].

The consumption frequency of certain food items was designated from the participants on a daily, weekly, or monthly basis by highly experienced dietitians [12]. The Iranian Food Composition Table (FCT) lacks complete data on the nutritional constitution of certain items; therefore, the US Department of Agriculture Food Composition Table (USDA FCT) was used for the major analyses. Meanwhile, items that were not listed in the USDA FCT were analyzed by the Iranian FCT [14], the trans-fat content was calculated by the McCance and Widdowson’s food composition table [15], and the nutrient content of mixed meals was accounted in accordance with the usual restaurant recipes.

### Biochemical measurements

Blood samples were collected at baseline and follow-up after an overnight fasting. The levels of triglyceride (TG) and fasting serum glucose (FSG) were measured by enzymatic colorimetric using phosphate oxidase and glucose oxidase, respectively. Similarly, the post-precipitated apolipoprotein B-containing lipoproteins along with phosphotungstic acid, were used to designate the HDL-c. The analyses were conducted by a Selectra 2 auto-analyzer (Vital Scientific; Spankeren, Netherlands) and the Pars Azmoon kits (Pars Azmoon Inc.; Tehran, Iran).

### Definition of terms

The risk score of CVD was calculated by the “general CVD” algorithms specified for each gender. This algorithm took the TC, HDL-C, age, SBP, hypertension medication, smoking, and diabetes into account [16]. The definition of diabetes itself included 2-h serum glucose above 200 mg/dL, fasting serum glucose above 126 mg/dL, or treatment with anti-diabetic medications [17]. Similarly, hypertension was defined as SBP higher than 140 mmHg, DBP higher or equal to 90 mmHg, or administration with hypotensive medications [18].

### Definition of outcome

Annual telephone call follow-ups were used to collect information regarding possible medical events by a physician or a trained nurse. Additional data were retrieved from medical records. An adjudication committee consisting of a physician, an internist, an epidemiologist, a cardiologist, and an endocrinologist would review the obtained data, and the final diagnosis was confirmed by a predefined coding protocol. Other experts were invited to the committee as needed [19].

CVD was primarily described as any history of coronary heart disease (CHD) or definite fatal CHD, definite fatal and non- fatal stroke, or CVD mortality [20]. CHD terminology alone included any cases of definite myocardial infarction (MI), probable MI, unstable angina pectoris, and sudden cardiac death [21]. Collecting data regarding the CVD-related outcomes are described in detail elsewhere [12].

Taken together, in the present study, CVD definition was based on any history of CHD or definite fatal CHD, definite fatal and non- fatal stroke, or CVD mortality. Since the main purpose of the current study was to address the risk of CVD outcomes related to the dietary fat composition at the end of the follow-up period, it worth mentioning that none of the participants had any history of CVD-related outcomes at the baseline.

### Statistical analysis

Data analyses were performed by IBM SPSS version 20.0 (SPSS Inc., Chicago, IL, USA) and *p*-value < 0.05 were considered as statistically significant. To compare the frequency and mean of the baseline characteristics across tertiles of total fat intake, univariate analysis, and chi-square tests were used. The significant differences in the frequency (%) of qualitative variables were detected by the chi-square test. Correspondingly, the CVD incidence score, total dietary fiber, and energy intake were considered as the cofounding variables of this one-way analysis (P_E_ < 0.2). Due to the highly significant value of the physical activity factor (P_E_ > 0.2), it was not ultimately selected for the final multivariable model.

To compute the 95% confidence intervals (CIs), and the Hazard ratios (HRs), the Cox proportional hazards regression model was used. The potentially confounding variables were adjusted in three models to estimate the HRs of CVD incidence risk across the tertiles of total fat, SFA, MUFA, PUFA, oleic acid, omega 6/omega 3 (ω-6/ω-3) ratio, and eicosapentaenoic acid + docosahexaenoic acid (EPA + DHA). In the first model, no adjustment was applied for the variables. For the CVD risk score, the second model was adjusted for TC, type 2 diabetes, smoking, age, HDL-c status, and hypertension treatment, and the calculations relied on the sex-specific “general CVD” algorithms [16]. Subsequently, the CVD score validation was evaluated among the Iranian population, which, in the case of our study, is also the main predictor of CVD events [22]. The third model was adjusted for total dietary fiber and energy intake. The Cox proportional hazard regression model used the median values of total fat, SFA, MUFA, PUFA, oleic acid, ω-6/ω-3 ratio, and EPA + DHA as continuous variables in the assessment of the overall HRs trend across the tertiles. The concept of time to event was used to describe the onset of an event or time to the completion of the follow-up.

## Results

In the current study, 2369 participants (1030 men and 1384 women) were recruited. At baseline, the mean age and BMI of the participants were 38.5 ± 12.7 years and 26.6 ± 4.8 kg/m^2^, respectively. Within a follow-up period of 6.7 ± 1.4 years, 79 cases of CVD events were detected, and angiographic proven CVD (40.4%), definite MI (24.6%), unstable angina (12.2%), and stroke (8.8%) were the most common. Approximately 30.1, 10.6, and 9.6% of the total energy consumption were contributed to dietary fat (76.2 g), MUFA (26.2 g), and oleic acid (23.6 g), respectively. Almost 90% of dietary MUFA was driven from oleic acid resources.

The distribution of CVD common risk factors among patients diagnosed with CVD and those free of CVD at baseline (2006–2008) is described in Table 1. Generally, diabetes (13.2 vs. 3.7%, *P* = 0.001) and hypertension (42.1 vs. 9.4%, *P* = 0.001) tended to be more prevalent among those diagnosed with CVD-related outcomes. Also, at baseline, the CVD patients appeared older and were more likely to have higher BMI, WC, FPG, TG, and blood pressure levels as well as CVD risk score (*P* < 0.05). Also, no significant difference was observed concerning the physical activity and the proportion of the inactive individuals between the two groups.

**Table 1:** Baseline characteristics of the participants

	Participants with CVD outcomes (*n* = 79)	Participants without CVD outcomes (*n* = 2290)	*P* value
Age (y)	58.4 ± 9.7	37.4 ± 12.8	0.001
Male (%)	68.4	42.6	0.001
Smoking (%)	20.2	11.7	0.024
Physical activity (MET/h-min)	30.1 ± 46.1	35.0 ± 56.7	0.35
Low-physical activity (%)	40.5	38.7	0.41
Body mass index (m^2^/kg)	28.4 ± 4.4	26.5 ± 4.8	0.005
Waist circumference (cm)	97.4 ± 9.9	87.9 ± 13.3	0.001
Serum creatinine (mmol/L)	102 ± 24.1	91.8 ± 13.1	0.001
Systolic blood pressure (mm Hg)	128 ± 19.0	109 ± 14.8	0.001
Diastolic blood pressure (mm Hg)	79.9 ± 11.2	72.4 ± 10.3	0.001
Fasting blood glucose (mg/dL)	104 ± 37.5	88.3 ± 16.1	0.001
Serum triglycerides (mg/dL)	188 ± 102	132 ± 77.4	0.001
High Density Lipoprotein Cholesterol (mg/dL)	39.4 ± 7.6	43.3 ± 10.3	0.001
Diabetes (%)	13.2	3.7	0.001
Hypertension (%)	42.1	9.4	0.001
Cardiovascular diseases risk score	20.1 ± 1.2	19.8 ± 0.6	0.004

Data are mean + SD (unless stated otherwise)

Baseline characteristics and dietary intake of TLGS studied population after the follow-up and across tertiles of total fat intake are described in Table 2. Motivated by the results, participants with lower consumption of fat resources tended to be older, and their physical activity was higher across all tertiles. Furthermore, participants on the upper tertiles of MUFA intake were more likely to be affiliated with greater consumption of total energy, total fat, SFA, PUFA, oleic acid, linoleic acid, and alpha-linolenic acid among the tertiles. In contrast, the BMI, FBS, HDL-c, LDL, TG, DBP, and EPA + DHA factors across tertiles of MUFA were insignificant.

**Table 2:** Baseline characteristics of the study participants across tertiles of dietary total fat intakes

	Tertiles of dietary total fat intakes			
	T 1	T 2	T 3	P for trend
Age (years)	40.6 ± 13.2	38.3 ± 12.5	36.3 ± 12.2	0.001
Male (%)	43.1	38.5	47.5	0.004
Physical activity (METS/wk)	33.9 ± 55.4	36.7 ± 55.4	37.1 ± 55.1	0.584
BMI (kg/m ^2^)	26.9 ± 5.1	26.5 ± 4.9	26.4 ± 4.6	0.057
FBS (mg/dl)	88.8 ± 19.2	88.5 ± 17.1	88.2 ± 16.1	0.159
HDL-c (mg/dl)	42.6 ± 9.8	43.4 ± 10.7	43.5 ± 10.6	0.182
LDL (mg/dl)	114 ± 31.8	113 ± 33.2	110 ± 29.5	0.088
TG (mg/dl)	137.1 ± 80.4	132.5 ± 80.7	131.3 ± 76.1	0.320
SBP (mmHg)	110 ± 16.1	108 ± 14.5	109 ± 15.6	0.033
DBP (mmHg)	73.1 ± 10.3	72.4 ± 10.2	72.6 ± 10.7	0.349
Total energy (kcal/d)	1640 ± 436	2332 ± 630	2808 ± 530	0.001
Total fat	27.5 ± 5.9	29.9 ± 6.4	34.8 ± 5.9	0.001
SFA	9.2 ± 2.5	10.3 ± 2.7	11.1 ± 2.6	0.001
MUFA	9.5 ± 2.5	10.4 ± 2.5	11.9 ± 2.5	0.001
PUFA	5.6 ± 1.9	6.3 ± 2.1	6.8 ± 1.9	0.001
Oleic acid	8.5 ± 2.4	9.5 ± 2.4	10.7 ± 2.4	0.001
Linoleic acid	4.8 ± 1.8	5.5 ± 1.8	5.8 ± 1.8	0.001
Alfa-linolenic acid	0.38 ± 0.1	0.45 ± 0.1	0.50 ± 0.1	0.001
EPA + DHA	0.05 ± 0.07	0.06 ± 0.22	0.04 ± 0.06	0.301

Data are mean + SD (unless stated otherwise)

*BMI* Body mass index, *FBS* Fasting blood glucose, *HDL-c* High-density lipoprotein cholesterol, *LDL* Low-density lipoprotein cholesterol, *TG* Triglyceride, *SBP* Systolic blood pressure, *DBP* Diastolic blood pressure, *SFA* Saturated fatty acid, *MUFA* Monounsaturated fatty acid, *PUFA* Polyunsaturated fatty acid, *EPA + DHA* Eicosapentaenoic acid + docosahexaenoic acid

The HR of the CVD incidence (95% CIs) across the tertiles of dietary fat constitutions is described in Table 3. Dietary consumption of total fat (HR = 0.55, 95% CI = 0.32–13 0.95; *P* = 0.030) and SFA (HR = 0.48, 95% CI = 0.27–0.83; *P* = 0.008) were inversely associated with the incidence of CVD events in the crude models; nevertheless, after adjusting for potential confounders, no association was observed. In contrast, the consumption of dietary MUFA resources was negatively associated with the incidence of CVD (HR = 0.26, 95% CI = 0.09–0.74; P trend = 0.012).

**Table 3:** Hazard ratios (95% CI) of cardiovascular disease across tertiles of dietary fats

	Tertiles of dietary fat intakes			
	T 1	T 2	T 3	P for trend
Total fat				
Crude	1	0.56 (0.33–0.96)	0.55 (0.32–0.95)	0.030
Model 1	1	0.59 (0.34–1.02)	0.63 (0.37–1.09)	0.094
Model 2	1	0.51 (0.27–0.97)	0.48 (0.22–1.02)	0.070
SFA				
Crude	1	0.53 (0.31–0.90)	0.48 (0.27–0.83)	0.008
Model 1	1	0.60 (0.35–1.03)	0.56 (0.32–0.99)	0.042
Model 2	1	0.55 (0.28–1.08)	0.50 (0.21–1.21)	0.147
PUFA				
Crude	1	0.87 (0.52–1.45)	0.63 (0.36–1.10)	0.111
Model 1	1	0.94 (0.55–1.59)	0.71 (0.40–1.27)	0.263
Model 2	1	0.96 (0.52–1.77)	0.78 (0.34–1.79)	0.579
MUFA				
Crude	1	0.58 (0.35–0.98)	0.42 (0.24–0.75)	0.003
Model 1	1	0.61 (0.36–1.03)	0.48 (0.27–0.85)	0.011
Model 2	1	0.45 (0.22–0.89)	0.26 (0.09–0.74)	0.012
Oleic acid				
Crude	1	0.51 (0.30–0.88)	0.44 (0.25–0.77)	0.003
Model 1	1	0.54 (0.31–0.94)	0.49 (0.28–0.87)	0.011
Model 2	1	0.41 (0.20–0.82)	0.30 (0.12–0.80)	0.016
EPA + DHA				
Crude	1	0.52 (0.30–0.89)	0.55 (0.32–0.94)	0.062
Model 1	1	0.50 (0.29–0.87)	0.54 (0.31–0.93)	0.062
Model 2	1	0.49 (0.28–0.86)	0.56 (0.32–0.97)	0.085
Omega-6/omega-3 ratio				
Crude	1	0.57 (0.33–0.99)	0.70 (0.41–1.17)	0.234
Model 1	1	0.53 (0.30–0.95)	0.74 (0.44–1.25)	0.357
Model 2	1	0.51 (0.29–0.91)	0.68 (0.40–1.16)	0.241

Cox proportional hazard regression models were used to estimate hazard ratios (HRs) with 95% confidence intervals (CIs) for cardiovascular diseases across tertiles of mono-unsaturated fatty acids, linoleic acid and omega-6/omega-3 ratio

Model 1: adjusted for CVD risk score

Model 2: additionally adjusted for energy intake, total fiber, total fat intake

*SFA* Saturated fatty acids, *PUFA* Polyunsaturated fatty acid, *MUFA* Monounsaturated fatty acid, *EPA + DHA* Eicosapentaenoic acid + docosahexaenoic acid

The risk of developing CVD events in the highest adjusted model of dietary MUFA and oleic acid (HR = 0.30, 95% CI = 0.12–0.80; *P* = 0.016) was higher against the lowest tertiles. Despite so, the association between greater load of EPA + DHA and decreased risk of CVD incidence was insignificant (HR = 0.56, 95% CI = 0.32–0.97; P trend = 0.085). Nevertheless, no statistically significant association was detected between the dietary PUFA and omega 6/ omega 3 intakes and the risk of CVD.

## Discussion

This study demonstrated that high loads of MUFA, oleic acid, and EPA+ DHA may possess promising cardioprotective effects, whereas total fat and SFA dietary intake were not significantly related to the CVD incidence risk. This new, albeit contradicting, finding calls for further focus on the quality and source of dietary fat, than merely the quantity and amount of total fat consumed.

According to the lipid-heart hypothesis [19], dietary fat must be consumed cautiously due to its effects on the lipid profile and cholesterol concentration [23–26]. More specifically, recommendations on the minimum intake of SFA and its association with CVD-related outcomes dominated nutritional guidelines for decades [27]. However, recent studies reported contradicting findings and have challenged the well-established restrictions and presumptions in this area. Within this context, an analysis on 347,747 subjects found no association between dietary intake of SFA and increased risk of CHD, CVD, or stroke [28]. This conclusion is in line with a recent meta-analysis of 9 cohorts [29] and a dose-response meta-analysis of 43 nested case-control studies [30]. Two separate studies in the framework of the Nurse’s Health Study (NHS) reported an insignificant association in a follow-up period over 20 years in this regard [31, 32]. Similarly, Chowdhury and colleagues did not clearly support guidelines that encourage diets enriched with PUFA and the lower SFA constitution [33], and Xu et al. have mentioned total fat, SFA, and MUFA as strong predictors of CHD mortality in American Indians [25]. In contrast, a 2015 study reported a lower risk of CVD and death among those in the highest quintile of total fat, MUFA, and PUFA intake comparing to those in the lowest quintile [34], which was supported by another study by Erkkila¨ and colleagues [23].

The findings of the present study concerning the dietary intake of MUFA were in agreement with previous investigations [4, 35]; however, our conclusion on the consumption of PUFA contradicted the presumptions [36, 37]. Oleic acid is the most prevalent form of MUFA in the human diet (∼90%) [32]. Guasch-Ferré and colleagues reported that higher intake of MUFA and PUFA reduced the incidence of CVD and CVD mortality [34]. Likewise, the highest quintile of PUFA had the lowest risk of CHD, a 32% decline in the NHS study [32]. A more recent follow-up of this study reported a close result of a 25% decline [31]. Delgado-Lista and colleagues reviewed 21 RCTs and found an overall reduction of 10% in the risk of CVD of any kind, a 9% decrease in risk of cardiac death, and an 18% decrease in coronary events [38]. This finding was supported by the Kuopio Ischemic Heart Disease Risk Factor Study [39] and the combined NHS and Health Professionals Follow-up cohort Study (HPFS) analysis [40].

A study that has analyzed 20 RCTs endorsed the protective effect of n-3 PUFA supplementation against CVD death [41]. However, a large-scale clinical trial reported no association between the daily n-3 fatty acids treatment and the reduction of CVD mortality and morbidity [42]. Despite the totality of the studies, some conflicting evidence has demonstrated that PUFA may increase the risk of CVD-related outcomes [43, 44], or possess no significant associations with the CVD risk [45].

The findings of the current study suggest that EPA + DHA can reduce the risk of CVD; however, it was not statistically significant. The observed cardioprotective effect of EPA + DHA is in agreement with the previous review papers and clinical trials [44, 46].

Several studies have investigated the association between dietary fats and fatty acids and the incidence of CVD. It is believed that the amount of fat consumption, regardless of the fatty acid composition, has little impact on cardiac health and vascular function [46]. Also, CVD has multiple interacting dietary determinants with the overall dietary patterns, including the dietary sources and the fatty acid composition [5].

Various prospective studies and clinical trials have favored substituting dietary SFA with MUFA and PUFA (with sufficient n-3 PUFA) [1, 5, 35, 47]. In this respect, a NHS study reported that total MUFA from both plant (MUFA-P) and animal resources (MUFA-A) could reduce mortality, yet MUFA-A was associated with a higher rate of CVD mortality [48]. Additionally, 22 years of follow-up in this study clarified that substituting SFA with MUFA-P could reduce the risk of CVD mortality [49].

The potential lipid-heart mechanisms can be attributed to the reduced production of inflammatory and reactive oxygen species by PUFA [50], inactivation of sterol regulatory element-binding protein (SREBP), increased expression of hepatic LDL receptor by MUFA [51], and higher levels of oxidative stress by SFA and trans fat [52]. Oleic acid, as the most crucial component of the MUFA group, has also been shown to decrease the LDL and TC levels [53].

The current study had some limitations and strengths. The major strengths included having a population-based and prospective design, high follow-up rate and duration, obtaining detailed information on the common risk factors and potential confounders, and using a validated FFQ to collect dietary intake data. The main Limitations was the limited control of the potential residual confounders, unavailability of MUFA components other than oleic acid, and unexplained and unmeasured confounders (i.e. genetic and serum-free fatty acids levels). Also, due to the potential modifications in the routine dietary habits and CVD risk factors of the participants over the study duration, certain misclassifications were unavoidable, which could have led to biased HRs towards the null. However, this issue is not far from expectations in the typical prospective studies. It worth noting that the present study was performed on a population with a relatively low incidence of CVD outcomes to make a definitive and firm conclusion. Therefore, further investigations on populations with higher rates of CVD events are required to obtain more accurate and profound results. Although the low number of CVD cases probably has affected the ultimate results, it is yet comparable to other Iranian populations [54].

## Conclusion

Dietary interventions are the primary strategies to prevent CVDs. In conclusion, this prospective study demonstrated that the fatty acid composition of different food sources influences CVDs incidence rate. A diet rich in MUFA, oleic acid, and EPA + DHA significantly reduced the risk of CVDs in the current study, whereas such association was not found for total fat, SFA, and PUFA. Caution should be taken when generalizing the results to populations with higher rates of CVDs, yet the findings can be generalized to other populations with identical dietary patterns. Since the incidence of CVDs is on rise worldwide, these investigations reveal the need for studies with larger samples and more extended follow-up periods.

## Data Availability

The database used and/ or analyzed during the current study available from the corresponding author on reasonable request.

## Abbreviations

CAD: Coronary Artery Disease
CHD: Coronary heart disease
CVD: Cardiovascular disease
DBP: Diastolic Blood Pressure
DHA: Docosahexaenoic acid
EPA: Eicosapentaenoic acid
FSG: Fasting Serum Glucose
HDL: High-density lipoprotein cholesterol
LDL: Low-density lipoprotein cholesterol
MUFA: Monounsaturated Fatty Acids
PUFA: Polyunsaturated Fatty Acids
SBP: Systolic Blood Pressure
SFA: Saturated Fatty Acids
TC: Total Cholesterol
TG: Triglyceride
TLGS: Tehran Lipid and Glucose Study
